# Economic fluctuations and cardiovascular diseases: A multiple-input time series analysis

**DOI:** 10.1371/journal.pone.0219358

**Published:** 2019-08-06

**Authors:** Chiachi Bonnie Lee, Chen-Mao Liao, Li-Hsin Peng, Chih-Ming Lin

**Affiliations:** 1 Department of Health Services Administration, College of Public Health, China Medical University, Taichung, Taiwan; 2 Department of Applied Statistics and Information Science, Ming Chuan University, Taoyuan, Taiwan; 3 Department of Healthcare Information and Management, Ming Chuan University, Taoyuan, Taiwan; Xiamen University, CHINA

## Abstract

**Objectives:**

Little is known about the gender and age differences associated with the effects of economic fluctuations on hospitalization for cardiovascular diseases. This paper investigates the impact of economic fluctuations on hospitalization for ischemic heart disease (IHD), stroke, and hypertension by age and gender between January 1996 and December 2012 in Taiwan.

**Methods:**

We adopted a multiple-input time series analysis to examine the strength of the immediate and latent effects of the 17-year quarterly unemployment rates (UR), air pollution exposure (APE), gross domestic product (GDP), per capita consumption expenditure in cigarette and alcohol (ECA), and per capita healthcare expenditure (HE) on the adjusted quarterly incidence rate of hospitalization. The data used in this paper were retrieved from the National Health Insurance Research Database and the website of the Directorate-General of Budget, Accounting and Statistics (DGBAS), Executive Yuan.

**Results:**

Our findings indicate that higher UR increased IHD hospitalization in young men and women and middle-aged women but reduced stroke hospitalization in young men. Higher APE increased IHD hospitalization in young men but reduced it for young women, increased stroke hospitalization in old men and middle-aged women but reduced it for young men, and increased hypertension hospitalization in middle-aged men and young women. Higher ECA reduced IHD hospitalization in middle-aged men, increased stoke hospitalization in middle-aged and old men and middle-aged women. Higher HE reduced IHD hospitalization in old men, young and old women, reduced stroke hospitalization in old women, and reduced hypertension hospitalization in young and middle-aged women.

**Conclusions:**

Overall, we found that the economic fluctuations caused increased harmful effects in certain population subgroups but also brought some soothing effects to some groups.

## Introduction

Cardiovascular diseases (CVDs) account for approximately 31% of all global deaths [1], and various research studies have investigated the association between cardiovascular mortality and economic fluctuations. Procyclical effects (where such an effect is defined as a decrease in the unemployment rate predicting an increase in mortality due to cardiovascular diseases and vice versa) were found in 23 OECD (Organization for Economic Cooperation and Development) countries [2], the USA [3–6], and 8 Asia-Pacific countries [7]. However, another study found that the relationship between cardiovascular mortality and economic fluctuations may not be procyclical and may even have been countercyclical for certain age groups in the USA from 1961 to 2010 [6]. Countercyclical effects (where such an effect is defined as a decrease in the unemployment rate predicting a decrease in mortality due to CVDs and acute myocardial infarction and vice versa) were also found in Sweden for those of prime working age, that is, between 20 and 49 years old [8], and in Greece [9]. However, only examining the mortality effects of society-wide economic conditions may understate the overall effects of those conditions on cardiovascular health [10, 11], while investigating the morbidity effects of economic fluctuations could contribute to the development of preventive strategies to redress the adverse effects of economic fluctuations on CVDs.

The morbidity effects of economic fluctuations on CVDs remain inconclusive. Procyclical effects were found in the USA [4, 12] and Mexico [13], while countercyclical effects were found in a systematic review [14] and in Sweden [8]. The review found that higher per capita gross domestic product (GDP) adjusted for purchasing power parity and total health expenditures per capita at purchasing power parity were related to lower incident risk of stroke [14]. Svensson [8] found that a one percentage point decrease in the unemployment rate decreased the incidence of acute myocardial infarction for individuals in Sweden aged 20–49 years by 3.2%. However, no significant association was found in Iceland [15] or in Greece [16].

Whether their effects are procyclical or countercyclical, it is clear that economic fluctuations can influence the incidence of cardiovascular diseases. In the procyclical pathway, it has been suggested that economic growth might bring adverse effects through worse air pollution [17, 18]; increased consumption of saturated fats, alcohol, and tobacco [19, 20], and long working hours [21, 22]. On the other hand, economic growth could have beneficial effects on heart health by increasing budgets for national health expenditures and vice versa. In Switzerland, the higher the real cantonal GDP per capita was, the greater the public health care expenditures were [23]. In Greece, the health spending growth declined much more than that of GDP during the Great Recession [24].

Further attention should be paid to gender and age differences in cardiovascular diseases as a result of economic fluctuations. The male CVD mortality and morbidity rates were higher than those for women (335 per 100,000 for men and 242 per 100,000 for women in mortality; 6,833 per 100,000 for men and 5, 812 per 100,000 for women in prevalence) in 2015 [25]. Nevertheless, CVD causes approximately 1⁄3 of all female deaths worldwide, and female CVD prevention and management merit further attention [26]. A study in the US found that the absolute decline in cardiovascular disease mortality due to the Great Recession was slightly greater among women than men from 2005 to 2010 [5]. Similarly, a study in Sweden found that women experienced a greater share of the decreased incidence and mortality from acute myocardial infarction (AMI) due to economic growth during the period from 1987 to 2003 [8]. Relatedly, the World Heart Federation has highlighted the need for the analysis of gender differences to combat gender inequality [27].

Furthermore, economic fluctuations could have different effects on people of different ages. In the US, increases in the unemployment rate were found to have reduced deaths from CVDs among adults aged 65 years and above [5], as well as among those aged 85 years and above [6]. A study conducted in the US found that a one percentage point fall in unemployment was related to 4.3, 13.3, and 12.8% increases in the prevalence of ischemic heart problems for the full study sample, 30–64-year-old employed individuals, and working men aged 30 to 55 years [12]. Conversely, a Swedish study found a countercyclical effect for those of prime working age, that is, between 20 and 49 years of age, in that a one percentage point increase in the unemployment rate increased the heart attack mortality and morbidity rates [8]. At the individual level, a Swedish cohort study found an increased risk of hospitalization due to stroke for men aged 35–49 years following job loss [28]. In light of these differential effects on various socio-economic groups, further inquiries addressing the differential effects on health outcomes will help identify the most vulnerable subgroups, thus allowing for the development of effective prevention strategies [29].

In Taiwan, cardiovascular diseases were the second leading cause of deaths in 2016[30]. In related research, however, the focus has been placed on the impacts of clinical symptoms[31], lifestyles[32], and the introduction of new recognition criteria for overwork [33]. Little attention has been paid, meanwhile, to the association between macroeconomic activities and CVDs in Taiwan. Nevertheless, Taiwan is relatively vulnerable to global economic fluctuations because it relies heavily on exports[34]. During the period from 1997 to 2016, the unemployment rate in Taiwan varied between 2.03% and 6.13%[35], while GDP growth rates varied between -7.65% and 12.46%[34]. Meanwhile, Taiwan reported much longer annual working hours (with an average of 2,104 hours) than the OECD countries (with an average of 1,751 hours) in 2015[36, 37]. Therefore, this study, using a multiple-input time series analysis, sought to examine the association between CVDs and macroeconomic indicators in Taiwan from 1996 to 2012, and further investigated whether the effects on different socioeconomic subgroups in Taiwan were immediate or latent.

## Methods

### Data source and dependent variables

Our sample period was 1996–2012, with quarters being the unit of observation. The dependent variables were the age-specific incidence rates (new cases per 100,000) of hospitalization due to cardiovascular and cerebrovascular diseases. Data were retrieved from the National Health Insurance Research Database (NHIRD), for which claims data from approximately 99% of the public and private hospitals that are contracted with the National Health Insurance Program (NHIP) in Taiwan are collated. Taiwan launched the single-payer NHIP in March of 1995. The National Health Insurance Administration (NHIA) sponsored the creation of the NHIRD to house the data from the NHIP. The National Health Research Institute (NHRI), a not-for-profit institute entrusted with maintaining the NHI data, then releases the NHIRD to academics for research purposes. The specific data used in this study were retrieved from three NHIP research databases, namely, the “registry for beneficiaries”, the “inpatient expenditures by admissions” database and the “monthly claims summary for inpatient claims” database, were analyzed. The following information was obtained from the relevant health certificates included in the three claims datasets: scrambled personal identification number (PIN), gender, cause of disease, date of birth, and date of admission.

The primary diagnoses for each admission are coded in the NHIRD database according to the International Classification of Diseases, Ninth Revision, Clinical Modification (ICD-9-CM) codes. The number of admissions was counted on a seasonal basis according to the admission date of hospitalization, i.e. January–March, April–June, July—September, and October–December. This datum was further stratified by sex and age for the whole population of Taiwan. We counted individuals who suffered from CVDs including ischemic heart disease (ICD-9-CM codes 410–414.99), stroke (430–434), and hypertension (400–405.99). Totals of 793,319 and 555,792 admissions for males and females, respectively, were found to be related to the above CVDs during the study period. We classified these patients by age into three sub-groups, i.e. young adults (aged 20–39 years), middle-aged (aged 40–64 years) and elderly (age ≥ 65 years). Populations were used as the denominator to calculate the age-sex-specific quarterly incidence rates of hospitalization from household registrations in Taiwan. Many factors on the supply side (i.e., the accessibility factors) will have impacts on the actual hospitalization rates, such as changes in health policies, the *point value* under the global budget scheme, opening and closing of beds, or the outbreak of an epidemic such as the SARS epidemic in 2003. Therefore there is a need to adjust the incidence rate of hospitalization when comparing the rates with each other. As an outcome variable, each quarterly adjusted incidence rate of hospitalization (AIRH) due to CVDs was calculated further by division with an adjusted factor to avoid confounders from fluctuations in health policies or hospital service volumes over the 17-year period. The adjustment factor was obtained by dividing the all-cause hospitalization rate of each period with that of the first season of 2011, and this adjustment factor was then applied to the incidence rates of hospitalization, and the AIRHs were then stratified with regards to sex and age.

### Data source and independent variables

The quarterly gross domestic product (GDP), age-sex-specific unemployment rate (UE), per capita consumption expenditures on cigarettes and alcohol (ECA), and per capita healthcare expenditures (HE) over the 17 years, data for which were released by the Directorate-General of Budget, Accounting and Statistics (DGBAS), Executive Yuan, were viewed as potential factors. Except for the UN (unemployment rates, which were presented as percentages), the other potential factors were presented in US dollars and were deflated by the price of 2011. Daily air measurements collected from 75 air monitoring stations in Taiwan were used to calculate the pollution standard index (PSI) by converting the highest concentrations of 5 critical air pollutants (i.e. CO, SO_2_, O_3_, NO_2_, and PM). Based on Environmental Protection Agency (EPA) air quality standards, the daily PSI scale runs from 0 to 500 and is categorized into several groups based on the pollutant health effects: 0 to 100 = good to moderate, and above 100 = unhealthy to hazardous. The quarterly station-days with PSI above 100 data over the 17-year period, which were released by the EPA, were also collected and analyzed as a potential factor and as a surrogate of air pollution exposure (APE).

### Statistical methods

The study period began in January 1996 and ended in December 2012. The potential factors for each of those 68 quarters were also examined, as were those same factors at one and two quarters prior to each quarterly observation of the age-sex-specific incident rates of CVDs of interest. Conventionally, vector autoregression (VAR) is a stochastic process model used to capture the linear interdependencies among multiple time series. The VAR is established through the cross correlation function method, as proposed by Box and Jenkins in the 1970s [38]. The method is a major macroeconometric framework that has been extensively used for multivariate time series analysis [39]. In addition, the transfer function model (TFM) constructed by corner method, proposed by Liu and Hanssens in 1982 [40], can be applied to the model with multivariate series, and can estimate the transfer function and the noise terms in model less complicatedly. Due to the short length of the sample period in our study, it can be hard to estimate the several parameters of VAR in the case of multivariate model. We therefore adopted the TFM with corner method for our analysis. The stepwise forward method was also used to select the input series in the model, which may reduce the problem of collinearity raised by multiple input variables. The TFM we adopted included GDP, UN, ECA, HE, and APE as the input time series variables and each AIRH of CVDs as the output time series variable. Our purpose in using this TFM was to improve the output variable (i.e. dependent variable only) time series model by analyzing the dynamic relationship between the input time series data and the output time series data.

Using a two-variable input series as an example, let *Y*_*t*_ be the output series and *X*_1,*t*_,*X*_2,*t*_ be the input series, and also assuming that the noise *N*_*t*_ follows an *ARIMA*((*p*,*d*,*q*)(*P*,*D*,*Q*)_*s*_) model which is uncorrelated with *X*_1,*t*_,*X*_2,*t*_, we obtain the TFM as follows:

$$Y_{t}=\frac{w_{1}(B)}{\delta_{1}(B)}B^{b}X_{1,t}+\frac{w_{2}(B)}{\delta_{2}(B)}B^{b}X_{2,t}+N_{t}$$

Where *w*(*B*) is the temporal effect, *δ*(*B*) is the gradual decay effect on output series, and the rational polynomial $\frac{w(B)}{\delta (B)}B^{b}$ is the impulse response weights between *Y*_*t*_ and *X*_*t*_,. The parameter *b* is the time lag of the input time series’ effects on the output series and *B* is the backward shift operator.

Construction of the TFM was an iterative process as the construction of the ARIMA model included identification, estimation, and diagnostic checking. In the identification step, we specified a low order for the noise series and used the nonlinear least square method to estimate the impulse response weights. Furthermore, we used the autocorrelation function (ACF), partial autocorrelation function (PACF), and Dicky-Fuller test to check the stationarity for the estimated noise series. If the noise series was not stationary, then difference the input and output series accordingly, and repeat the identification step. When the noise was stationary, we used the corner method to choose the orders (*b*,*r*,*h*) of the TFM. Next, the statistically appropriate models were selected using the stepwise forward method and Akaike’s information criterion (AIC), with the latter being based on the maximum likelihood method (Bayesian Information Criterion was also tried and yielded similar results). If the model was adequate, we then pooled the identified transfer functions with the satisfied noise to estimate the parameters (*w*,*δ*) simultaneously. Finally, Box-Ljung Q statistics based on the residuals was used to diagnose whether the model was adequate. These methods were described in detail in two papers [41, 42] and a book [43]. SAS version 9.1 (SAS Institute Inc., Cary, NC, USA) was used for the storage and aggregation of the data, and all ARIMA analyses were performed with R version 3.2.5 (R Foundation for Statistical Computing, Vienna, Austria. 2016). The standard errors of the estimated coefficients were indicated directly and computed using the R package. According to the description of the R package, maximum likelihood and Hessian matrix methods are used to estimate the standard error.

### Ethics approval

The protocol for this study was approved by the Research Ethics Committee, National Taiwan University (NTU-REC 201512EM020). The dataset used in this study consisted of secondary data; all information was de-identified by data owners.

## Results

Table 1 summarizes the statistics of the key variables used in our analyses. Over the research period of 17 years, the average quarterly per capita GDP was USD3,750.85; the average per capita health expenditure (HE) was USD85.86; the per capita expenditure on cigarette and alcohol (ECA) was USD57.66; the unemployment rate (UE), which was further stratified by sex and age for later analysis, averaged 4.05%; and the air pollution exposure (APE) (PMI>100) was 218.60 station-days. The UE for people aged older than 65 years were not estimated, because most people of that age were retired. Table 2 lists the quarterly incidence rates (per 100,000) of hospitalization due to ischemic heart disease (IHD) (212.8), cerebrovascular disease (stroke) (70.6), and hypertension (71.8). Men had higher hospitalization rates for IHD and stroke, while women had a higher rate for hypertension. The incidence rates increased with age in both men and women, but they increased more significantly in women.

**Table 1:** Description of socioeconomic factors in Taiwan, 1996–2012.

Variables	Mean (SD)	Median	Minimum	1st quartile	3rd quartile	Maximum
GDP	3750.85 (800.45)	3526.79	2647.18	3093.71	4278.20	5591.73
HE	85.86 (22.17)	90.63	49.37	65.97	103.92	120.59
ECA	57.66 (7.75)	57.43	41.44	51.37	62.10	75.62
APE	218.60 (136.87)	192.00	29.00	120.75	270.50	641.00
UE	4.05 (0.99)	4.15	2.12	3.10	4.75	6.08
Men	4.41(1.14)	4.31	2.30	3.31	5.18	6.77
20–39	12.46 (2.98)	13.31	7.36	9.18	14.30	18.62
40–64	6.44 (2.07)	6.09	2.61	4.63	8.01	10.43
Women	3.55(0.86)	3.81	1.84	2.86	4.14	5.19
20–39	10.40 (3.26)	10.09	5.22	8.11	13.30	18.28
40–64	3.46 (1.42)	3.88	0.95	1.92	4.29	5.73

The socioeconomic factors included were GDP (gross domestic product; in USD), HE (per capita healthcare expenditures; in USD), ECA (per capita consumption expenditures on cigarettes and alcohol; in USD), APE (air pollution exposure; in station-days) and UE (sex-age-specific unemployment rate; as a %).

The UE for people aged older than 65 were not estimated, because most people of that age were retired.

SD: standard deviation.

**Table 2:** Quarterly incidence rates of hospitalization due to cardiovascular diseases (per 100,000 persons) in Taiwan, 1996–2012.

	Ischemic heart disease		Cerebrovascular disease		Hypertension	
	Mean (SD)	Median	Mean (SD)	Median	Mean (SD)	Median
Total	212.8 (236.7)	111.1	70.6(76.9)	42.3	71.8(102.9)	25.5
Men	255.5 (257.0)	158.1	76.0(71.7)	57.6	59.3(75.1)	24.7
20–39	6.3 (1.3)	6.0	4.9(0.6)	4.8	3.7(0.7)	3.8
40–64	158.1 (11.7)	158.1	59.9(16.5)	57.6	30.9(15.2)	24.7
> = 65	602.2 (72.7)	611.3	163.4(46.6)	152.9	143.2(76.0)	113.7
Women	170.2 (206.4)	66.0	65.2(81.6)	28.6	84.3(123.6)	26.2
20–39	1.4 (0.3)	1.4	2.1(0.3)	2.1	2.1(0.4)	2.1
40–64	64.6 (15.3)	66.0	30.6(10.6)	28.6	33.5(20.6)	26.2
> = 65	444.5 (110.8)	470.7	162.9(71.5)	142.6	217.3(135.6)	162.9

Based on the results from the maximum likelihood estimation of the final selected models with HE, ECA, APE, and UE as the input variables, Table 3 shows the significant effects of these input variables on the hospitalization rate for IHD. In terms of IHD, unemployment did take a toll on young men after six months, on young women after nine months, and on middle-aged women after six months (for each percentage increase in UE, there were 2.24%, 2.53%, and 1.95% increases in the IHD hospitalization rates for these three subgroups, respectively). Furthermore, seasonal composite effects of unemployment rate fluctuations were found in young men: since the effects of the two consecutive seasons nearly cancelled each out, no effect on the IHD hospitalization rate was observed if the UE was stable during the prior two and three periods. However, if the UE increased from low to high by 1%, then the IHD hospitalization rate increased by 5.05% (2.24 plus 2.81), and if the UE decreased from high to low by 1 percentage point, then the IHD hospitalization rate decreased by 5.05%. HE helped reduce the disease in older men after three months, in young women after six months, and in older women after three months (for each one US dollar increase in the HE, there were decreases in the IHD hospitalization rate by 0.29%, 0.70%, and 0.39% in these three subgroups, respectively). APE increased the IHD hospitalization rate in young men after six months (if APE increased by 1 station-day, then the IHD hospitalization rate increased by 0.47% after six months), while it decreased the IHD hospitalization rate in young women after three months. ECA did not appear to have negative effects on the studied groups in terms of the IHD hospitalization rate, and it even had the effect of decreasing the IHD hospitalization rate among middle-aged men.

**Table 3:** Effects of socioeconomic factors on incidence rates of hospitalization due to ischemic heart diseases in Taiwan.

Sex	Age	Selected variables^a^	b (lag)	ω^b^ (s.e.)	p-value	Mean (1/100,000)	Change (%)^c^ (95% CI)	Box-Ljung Q-statistics (df^d^) p-value
Men	20–39	UE	2	0.1398 (0.0593)	0.0183	6.25	2.24	15.5152 (12) 0.2145
							(0.3804, 4.0996)	
			3	-0.1756 (0.0603)	0.0036	6.25	-2.81	
							(-4.7010, -0.9190)	
		APE	2	0.0291 (0.0148)	0.0493	6.25	0.47	
							(0.0059, 0.9341)	
	40–64	ECA	2	-0.4209 (0.1521)	0.0057	158.12	-0.27	13.1196 (9) 0.1573
							(-0.4585, -0.0815)	
	> = 65	HE	1	-1.7711 (0.6616)	0.0074	602.20	-0.29	18.0828 (12) 0.1132
							(-0.5053, -0.0747)	
Women	20–39	APE	1	-0.0118 (0.0056)	0.0351	1.42	-0.83	13.5870 (8) 0.0932
							(-1.6030, -0.0570)	
		HE	2	-0.0099 (0.0018)	<0.0001	1.42	-0.70	
							(-0.9485, -0.4515)	
		UE	3	0.0359 (0.0145)	0.0133	1.42	2.53	
							(0.5286, 4.5314)	
	40–64	UE	2	1.2571 (0.4658)	0.007	64.55	1.95	16.6167 (10) 0.083
							(0.5356, 3.3644)	
	> = 65	HE	1	-1.7136 (0.4195)	<0.0001	444.52	-0.39	11.7236 (11) 0.3848
							(-0.5750, -0.2050)	

^a^ Variables were selected in a stepwise manner using the likelihood ratio test. The socioeconomic factors included were GDP (gross domestic product; in USD), HE (per capita healthcare expenditures; in USD), ECA (per capita consumption expenditures on cigarettes and alcohol; in USD), APE (air pollution exposure; in station-days) and UE (sex-age-specific unemployment rate; as a %).

^b^ Differences in the adjusted incidence rate of hospitalization due to ischemic heart diseases (1/100,000) were estimated with a quarterly autoregressive integrated moving average model.

^c^ The percentage changes and the 95% coefficient intervals (CI) in these changes were based on the mean of 68 quarterly observations.

^d^ Degree of freedom.

The effects of the same input variables on stroke and hypertension are shown in Tables 4 and 5, respectively. In terms of stroke, UE considerably reduced the hospitalization rate in young men after nine months, and HE had a positive effect on older women. ECA exerted an immediate (lag = 0) negative (increased) effect in middle-aged men, older men, and middle-aged women, yet its rippling impact (i.e. when lag>0) demonstrated opposing effects: while it increased the stroke hospitalization rate for middle-aged women (lag = 3), it reduced the stroke hospitalization rates for middle-aged men and older men (lag = 1). Furthermore, seasonal composite effects were found in the middle-aged men during period zero and one (for an ECA increase from low to high by 1 US dollar, the stroke hospitalization rate increased by 1.16%; for an ECA decrease from high to low by 1 US dollar, the stroke hospitalization rate decreased by 1.16%). Regarding the seasonal composite effects of ECA on the stroke hospitalization rate for older men, since the effects of the two consecutive seasons nearly cancelled each other out, no significant effect was found for the stable ECA. However, if the ECA increased from low to high by 1 US dollar, then the stroke hospitalization rate increased by 1.11%, and if the ECA decreased from high to low by 1 US dollar, then the stroke hospitalization rate increased by 1.11%. APE increased the immediate (lag = 0) stroke hospitalization rate in both old men and middle-aged women, but its rippling impact decreased the stroke hospitalization rate in young men (lag = 2). As for hypertension, Table 5 shows that HE only protected young and middle-aged women from hypertension (lag = 1), while air pollution had a negative effect on middle-aged men and young women.

**Table 4:** Effects of socioeconomic factors on incidence rates of hospitalization due to stroke in Taiwan.

Sex	Age	Selected variables^a^	b (lag)	ω^b^ (s.e.)	p-value	Mean (1/100,000)	Change (%)^c^ (95% CI)	Box-Ljung Q-statistics (df^d^) p-value
Men	20–39	UE	3	-0.1317(0.0514)	0.0104	4.88	-2.70	12.8750(12) 0.3782
							(-4.7644, -0.6356)	
		APE	2	-0.0354(0.0159)	0.0260	4.88	-0.73	
							(-1.3686, -0.0914)	
	40–64	ECA	0	0.1995(0.0896)	0.0260	59.87	0.33	11.4213 (11) 0.4087
							(0.0367, 0.6233)	
			1	-0.4996(0.0754)	<0.0001		-0.83	
							(-0.9205, -0.7395)	
	> = 65	APE	0	1.1421(0.3685)	0.0019	163.37	0.70	10.7095(10) 0.38056
							(0.2579, 1.1421)	
		ECA	0	0.9438(0.2364)	<0.0001	163.37	0.58	
							(0.2964, 0.8636)	
			1	-0.8722(0.3164)	0.0058		-0.53	
							(-0.9096, -0.1504)	
Women	20–39	APE	1	-0.0094(0.0092)	0.3069	2.13	-0.44	17.9934(14) 0.2071
							(-1.2866, 0.4066)	
	40–64	ECA	0	0.2875(0.0911)	0.0016	30.63	0.94	11.9358(10) 0.2894
							(0.3571, 1.5229)	
			3	0.1984(0.0781)	0.0111		0.65	
							(0.1502, 1.1498)	
		APE	0	0.1623(0.0759)	0.0325	30.63	0.53	
							(0.0443, 1.0157)	
	> = 65	HE	2	-0.7441(0.2887)	0.0099	162.85	-0.46	16.0795(11) 0.1382
							(-0.8075, -0.1125)	

^a^ Variables were selected in a stepwise manner using the likelihood ratio test. The socioeconomic factors included were GDP (gross domestic product; in USD), HE (per capita healthcare expenditures; in USD), ECA (per capita consumption expenditures on cigarettes and alcohol; in USD), APE (air pollution exposure; in station-days) and UE (sex-age-specific unemployment rate; as a %).

^b^ Differences in the adjusted incidence rate of hospitalization due to ischemic heart diseases (1/10^5^) were estimated with a quarterly autoregressive integrated moving average model.

^c^ The percentage changes and the 95% coefficient intervals (CI) in these changes were based on the mean of 68 quarterly observations.

^d^ Degree of freedom.

**Table 5:** Effects of socioeconomic factors on incidence rates of hospitalization due to hypertension in Taiwan.

Sex	Age	Selected variables^a^	b (lag)	ω^b^ (s.e.)	p-value	Mean (1/100,000)	Change (%)^c^ (95% CI)	Box-Ljung Q-statistics (df^d^) p-value
Men	20–39	HE	1	-0.0207(0.0142)	0.1449	3.74	-0.55	10.4034(11) 0.4945
							(-1.2942, 0.1942)	
	40–64	APE	0	0.0922(0.0390)	0.0181	30.94	0.30	13.6251(12) 0.3253
							(0.0529, 0.5471)	
	> = 65	HE	0	0.3288(0.3875)	0.1457	143.21	0.23	14.8242 (13) 0.3185
							(-0.3003, 0.7603)	
Women	20–39	HE	1	-0.0152(0.0067)	0.0233	2.09	-0.73	14.9670 (12) 0.2432
							(-1.3583, -0.1017)	
		APE	1	0.0543(0.0219)	0.0132	2.09	2.60	
							(0.5462, 4.6538)	
	40–64	HE	1	-0.1878(0.0717)	0.0088	33.51	-0.56	14.9409(10) 0.1342
							(-0.9794, -0.1406)	
	> = 65	HE	1	-0.5440(0.3739)	0.1457	217.33	-0.25	16.4870(11) 0.1240
							(-0.5872, 0.0872)	
			2	-0.2785(0.3732)	0.4555		-0.13	
							(-0.4666, 0.2066)	

^a^ Variables were selected in a stepwise manner using the likelihood ratio test. The socioeconomic factors included were GDP (gross domestic product; in USD), HE (per capita healthcare expenditures; in USD), ECA (per capita consumption expenditures on cigarettes and alcohol; in USD), APE (air pollution exposure; in station-days) and UE (sex-age-specific unemployment rate; as a %).

^b^ Differences in the adjusted incidence rate of hospitalization due to ischemic heart diseases (1/10^5^) were estimated with a quarterly autoregressive integrated moving average model.

^c^ The percentage changes and the 95% coefficient intervals (CI) in these changes were based on the mean of 68 quarterly observations.

^d^ Degree of freedom.

**Fig 1:**
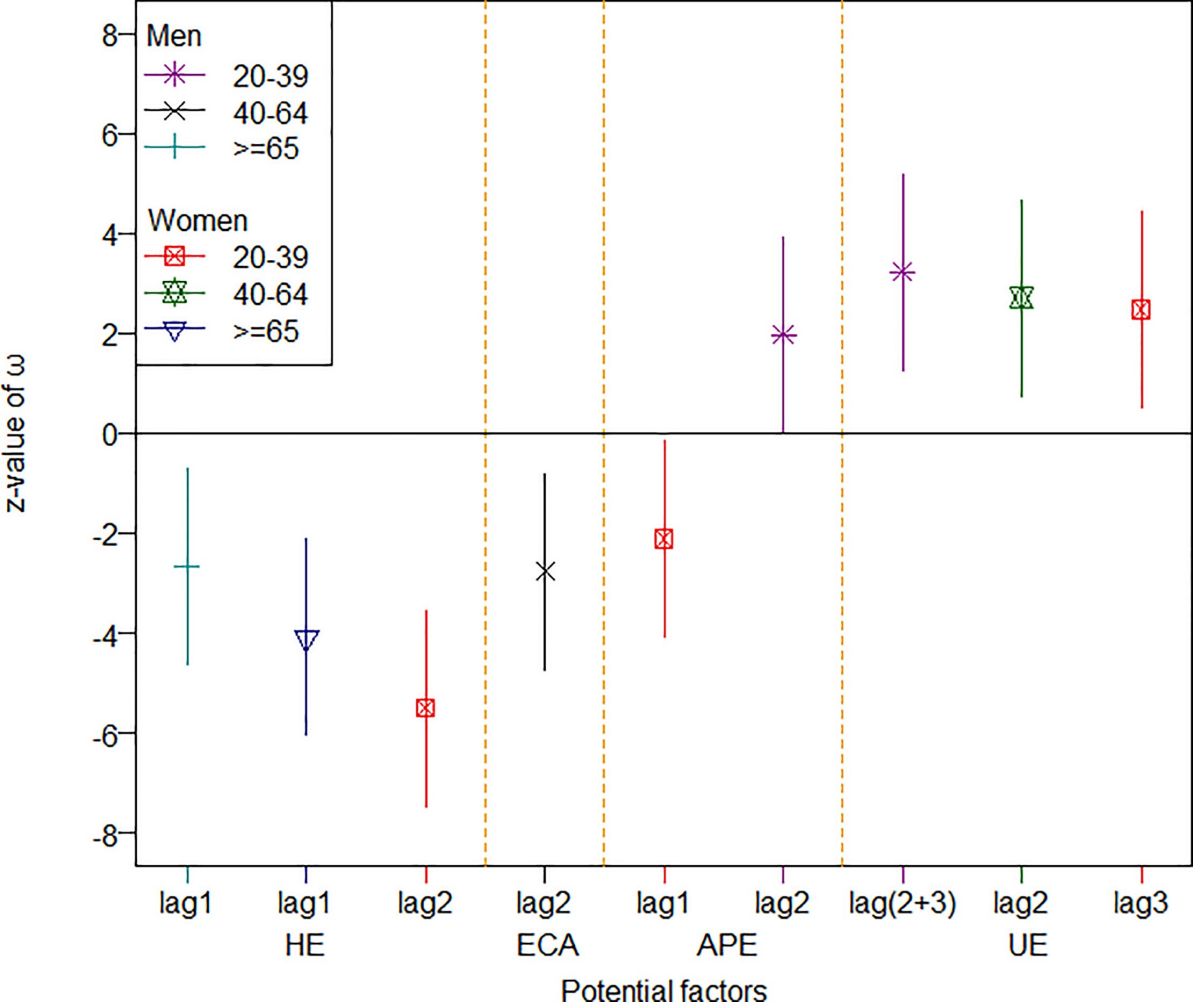
The effects of significant factors on incidence of ischemic heart diseases. The indicator and solid line present the z-value and 95% coefficient interval of ω, respectively; the lag with a bracket represents the integrated effect for two different lags with an increasing trend.

**Fig 2:**
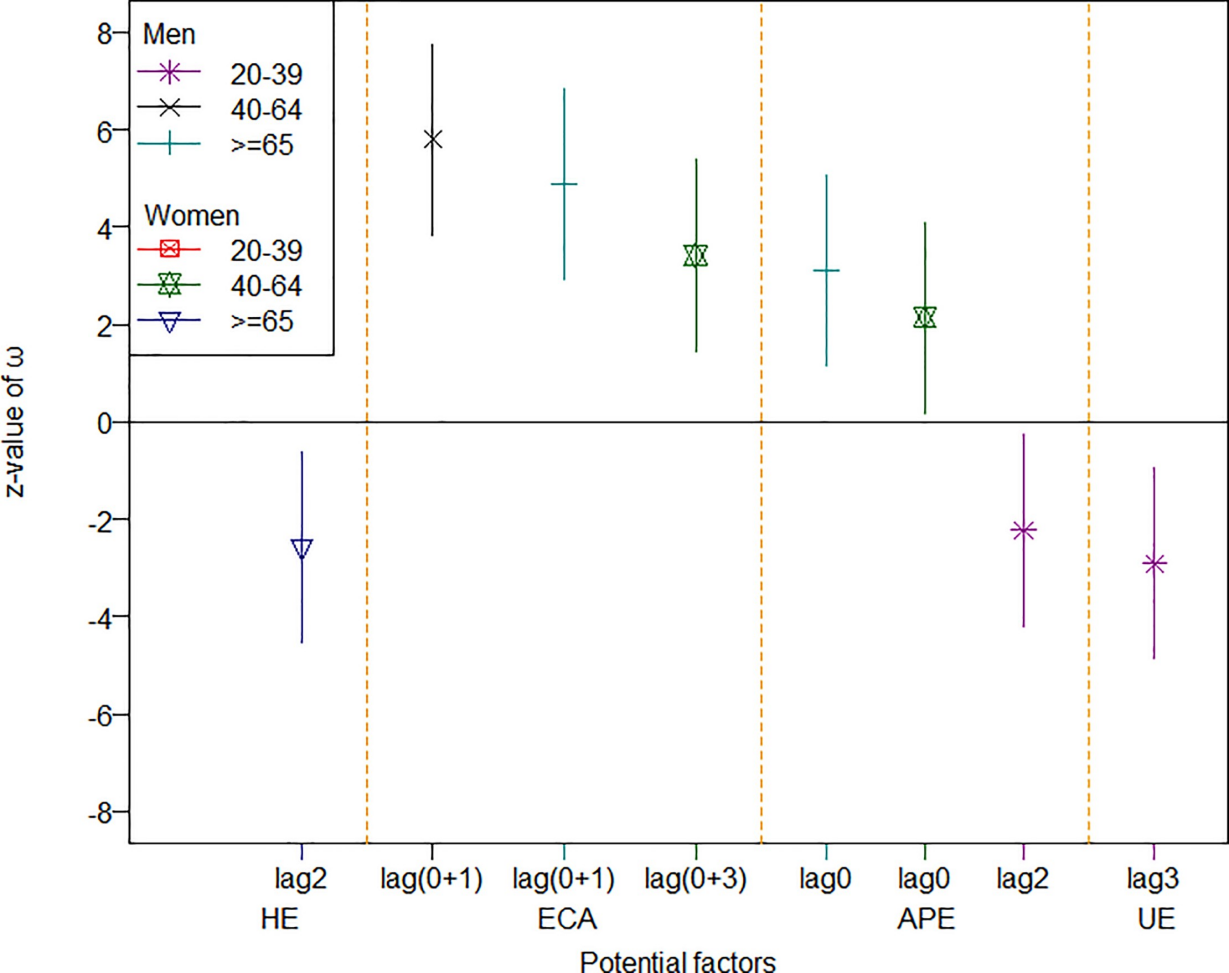
The effects of significant factors on incidence of stroke. The indicator and solid line present the z-value and 95% coefficient interval of ω, respectively; the lag with a bracket represents the integrated effect for two different lags with an increasing trend.

**Fig 3:**
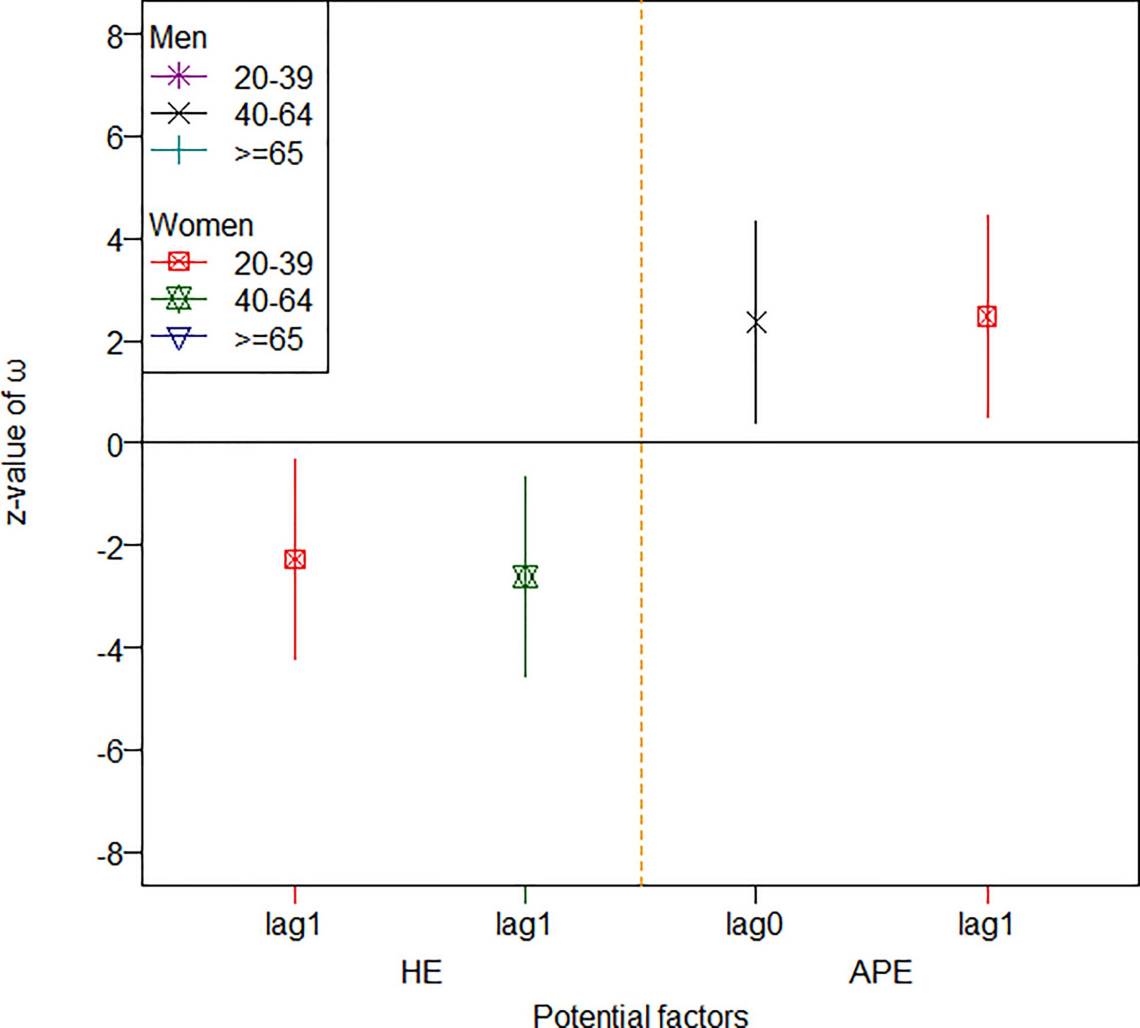
The effects of significant factors on incidence of hypertension. The indicator and solid line present the z-value and 95% coefficient interval of ω, respectively.

For easier reading and clearer presentation, Figs 1 to 3 summarize the standardized effects of the input variables on the three groups of diseases among the different subsets of the population (only the significant effects are shown). As shown in Fig 1, for an increase of one standard deviation in the unemployment rate, the hospitalization rates for IHD increased by somewhere between 2 to 4 standard deviations among young men, young women, and middle-aged women, and when HE increased by one standard deviation, the hospitalization rate for IHD decreased by as many as 2 to 6 standard deviations among old men, old women, and young women within one or two seasons. Fig 2 suggests that, for an increase of one standard deviation in air pollution (as measured by station-days), the stroke hospitalization rate increased by as many as around three standard deviations over the same season among old men and middle-aged women, while an increase of one standard deviation in the consumption of cigarette and alcohol could push the stroke hospitalization rate up by 4~5 standard deviations among old men and both middle-aged men and women. Furthermore, an increased unemployment rate could reduce the stroke hospitalization rate among young men, while old women responded positively to an increase in health expenditures. Fig 3 suggests that young and middle-aged women responded positively to increases in health expenditures, as the hospitalization rate for hypertension decreased by 2 standard deviations for an increase of one standard deviation in health expenditures. Furthermore, air pollution took tolls mainly on middle-aged men and young women in terms of the hospitalization rate due to hypertension.

## Discussion

To the best of our knowledge, this study is the first research to examine the impact of economic fluctuations on the hospitalization rates for CVDs by age and gender in Taiwan. This study identified specific vulnerable subgroups in Taiwan who suffered from negative impacts of economic fluctuations in terms of the hospitalization rates for ischemic heart diseases, stroke, and hypertension. At the same time, this study also identified subgroups who were inadvertently protected from the negative effects of economic fluctuations. By using the time series method with backward operators, this study is able to capture the effects of economic fluctuations in manifested or latent pathways on different demographic and gender subgroups in Taiwan.

A higher unemployment rate increased the IHD hospitalization rate in young men, young women, and middle-aged women after half a year; however, it decreased the stroke hospitalization rate in young men after nine months. Furthermore, a high unemployment rate is linked to job insecurity. In 2009 when Taiwan had the highest unemployment rates after the Great Recession of 2008, young men and women and middle-aged women might have experienced more job insecurity. Due to downsizing, the elimination of seasonal or temporary jobs, and mass-scale layoffs driven by personnel restructuring in the workplace, up to 57% of the laborers laid off in 2008 in Taiwan were young men and women [44]. Gender inequality in employment in Taiwan was pervasive. Middle-aged women (5.5% in 2009) were more likely to take on temporary or dispatched jobs than middle-aged man (4.1%) were, and the average monthly income of middle-aged women (NT$32,580) was 74% that of their male counterparts (NT$44,095) in 2009 [44]. Furthermore, women (5%) were more likely than man (0.7%) to encounter employment inequality due to the mismatch between their work and familial lives [45]. A systematic review and meta-analysis identified perceived job insecurity as a risk factor for incident IHD [46]. A study found that workers who reported job insecurity had significantly higher odds of health risk factors including being obese, sleeping less than 6 hours a day, and smoking every day [47]. These risk factors also contribute to the incidence of CVDs. This study further found that individuals facing job insecurity had a higher likelihood of developing serious mental illnesses within the past 30 days, with that likelihood being almost five times higher than the likelihood for those who were not dealing with job insecurity [47]. A Taiwanese study found that the 2008 economic recession led to increased rates of hospitalization for depressive illnesses in Taiwan, and the adjusted incidence rates of hospitalizations for depressive illnesses were generally higher in female subgroups than they were in male subgroups from 2007 to 2012, that is, both before and after the financial crisis [48]. Depression is significantly related to an increased risk of IHD [49]. This might partly explain the wider impact of the unemployment rate on young and middle-aged women. On the other hand, after nine months of unemployment, the unemployment rate was found to be negatively correlated to stroke-related hospitalization among young men in Taiwan. In Taiwan, a higher unemployment rate led to shorter working hours (r = -0.837, p < 0.01)[50], and a systematic review found that employees who worked long hours had a higher risk of stroke than those who worked standard hours [51]. This study did not find, meanwhile, whether the unemployment rate had either a positive or negative effect on people aged older than 65 years in Taiwan. This was because most people aged older than 65 years in Taiwan are retired, and only between 7.27% and 8.95% of them were still working between 1996 and 2012 [52].

Air pollution exposure (APE) increased the IHD hospitalization rate among young men, while it decreased the IHD hospitalization rate among young women. Hospitalizations for IHD were strongly related to short-term exposure to high levels of PM_10_ and PM_2.5_ during 2013–2014 in Shanghai, China, and that the effect of PMs was significantly greater in men than in women [53]. Perhaps young men are more likely to work outdoors and experience high levels of APE than young women are. APE varies across geographic regions, and the impact of APE on IHD hospitalization rate could also vary across geographic regions. A recent study in China revealed a positive association between an increase in PM_2.5_ and PM_10_ concentration levels and IHD admissions in northern China but no effects or negative associations in southern China [54]. Future studies should be conducted to confirm the existence of geographical differences in health effects with respect to air pollution, so as to clarify the association between air pollution and the IHD hospitalization rate. Furthermore, APE increased the immediate stroke hospitalization rate in both old men and middle-aged women (lag 0), but its rippling impact decreased the stroke hospitalization rate in young men (lag = 2). Findings regarding the impact of short-term exposure to air pollution on stroke admissions still remain inconsistent. A systematic review and meta-analysis found evidence of inconsistent and nonsignificant associations between air pollution and hospital admissions for ischemic or hemorrhagic stroke [55]; however, another systematic review and meta-analysis found a positive association with stroke hospitalizations [56]. The results of this study indicate that old men and middle-aged women are the vulnerable subgroups in Taiwan. Most of the related studies have focused on older people, middle-aged and older people above, or people of all ages. Little is known, meanwhile, about the long-term impacts of APE on stroke hospitalization rates for young people of both genders. Further research is warranted to explore the mechanism or mechanisms underlying the impacts of APE on stroke hospitalization rates for young people. Moreover, the results of this study draw attention to a negative effect of APE on the hypertension hospitalization rates for middle-aged men and young women. A recent systematic review and meta-analysis indicated that ambient air pollution is positively related to increased blood pressure (BP) and hypertension [57]. Another study found that elevated concentrations of pollutants increase hospital admissions for hypertensive disorders and also that elevated PM levels increase BP in vulnerable subsets of individuals (such as pregnant women) [58].

Per capita consumption expenditures on cigarettes and alcohol (ECA) exerted negative impacts on the stroke hospitalization rates of middle-aged men, older men, and middle-aged women. A meta-analysis identified the pooled relative risks of stroke faced by male and female smokers [59], and another meta-analysis found that heavy alcohol consumption raises the relative risk for any kind of stroke [60]. This study revealed the seasonal composite effects of ECA on middle-aged and older men, that is, if the ECA decreased relative to the previous season, and increases in the current season, the incidence rates of hospitalization due to stroke will be compounded to 1.16% and 1.11% for middle-aged and old men, respectively. In Taiwan, male adult smoking rates (55.1% in 1996, 32.7% in 2012) have historically been much higher than those for females (3.3% in 1996 and 4.3% in 2012) [61]. A recent case-cohort study involving eight European countries found that alcohol intake was positively related to the risk of non-fatal stroke but inversely related to non-fatal IHD [62]. Similarly, this study found decreases in the risk of IHD hospitalization due to increases in ECA in middle-aged men. A small amount of wine or beer that is rich in polyphenols appears to have certain moderating effects that neutralize the otherwise negative effects of alcohol[63]. Oxidative stress might be compounded with smoking as a risk factor for cardiovascular diseases, and that the quantity of cigarettes smoked, not just smoking itself, matters in increasing the level of oxidative damage[64]. In this study, increased ECA was not found to be related to increased IHD hospitalization rates. This might be because polyphenols in alcohol provide protective effects that counteract the harmful effects of cigarettes, a possibility that further suggests that there may be a different mechanisms underlying the effects on IHD and stroke.

Higher health expenditures (HE) reduced the risks of IHD in older men, older women, and young women; reduced the risk of stroke in older women; and reduced the risks of hypertension in young and middle-aged women after three or six months. Taiwan implemented the NHIP in 1995, and the annual increase in the rate of national health expenditures ranged between 1.19% and 9.81% during the period covered by this study. In a study that examined 27 European nations, it was found that social insurance systems were less susceptible to austerity policies and tax revenue fluctuations than tax-flnanced healthcare systems between 1995 and 2011[65]. Taiwan’s NHIP is a single-payer social insurance system, and the annual increase in the rate of national health expenditures remained positive from 2001 to 2009, in spite of the negative GDP growth rates in those years [66]. As such, these studies together suggest that it is worthwhile to invest in health expenditures in order to protect people from cardiovascular diseases. This protective effect was more evident in women than men in Taiwan.

While theoretically, it is possible that our model can be affected under certain circumstances by endogeneity problems, such as possibility of hospitalization having some impact on our input variables. However, based on the existing literature, we were unable to demonstrate that increases or decreases in hospitalization lead to changes in air pollution or GDP. A patient may cut down on their short-term consumption of cigarette and alcohol during hospitalization, but it takes further evidence to determine whether the addicted patient would reduce ECA after being discharged from the hospital. The HE used in our study included out-of-pocket items such as nutrition supplements, and therefore, it is possible that the increase in hospitalization can lead to a higher HE during or after hospitalization if a patient increases his or her use of these items during or after hospitalization. We did not proceed any further with these analyses. TFM does not assess if the current values of the input variable are dependent on the past or current levels of the output variable for specific patterns. Thus, in contrast to its relevance in the investigation of causal relationships, our findings are less applicable to the use of input variables to forecast output variables.

## Strengths and limitations

One of the strengths of the time series methods applied in this study is that, by using the backward operator, these methods can summarize cumulative or composite effects across more than one period. This is not unlike the phenomena of *wave interference* in physics, that is, if the effects of a factor go in opposite directions in consecutive periods, than the effects on the current period will be either compounded to make a “wave” with a higher “amplitude”, or cancel each other out, depending on the patterns of the fluctuations in the previous periods. This speaks to the important fact that it is the fluctuations, not just the trend, nor the stationary state, of the economy or other environmental factors that have pronounced effects on people’s health—a point that this study can contribute to the literature. Additionally, this study fills the research gap regarding how economic fluctuations interact with gender and age to influence hospitalizations for cardiovascular diseases. Meanwhile, one major limitation of this study is that it failed to account for individual-level factors, such as lifestyles, which might have caused CVDs. Data regarding such factors, however, are not available in the investigated claims data. Future analyses could integrate both macroeconomic activities and individual-level factors affecting CVDs to refine our model. Furthermore, this study was supported by a grant and utilized chargeable data from the National Health Insurance Research Database. Due to a restricted budget, we were unable to extend the sample period beyond 2012. Further studies involving post-2012 data can be conducted to explore whether our findings remain relevant beyond 2012. Finally, this study utilized the differences in the time series until stationarity was reached, although it should be pointed out that the exact nature of the trend in nonstationary data is difficult to determine since even unit root tests may lack sufficient power to distinguish between stochastic trends and deterministic trends.

## Conclusion

In conclusion, the health care data for Taiwan from 1996 to 2012 indicate that middle-aged women are vulnerable, in terms of their susceptibility to hospitalization for ischemic heart disease and stroke, to unemployment, cigarette and alcohol consumption, and air pollution exposure. Middle-aged and old men are vulnerable, in terms of their susceptibility to hospitalization for hypertension and stroke, to cigarette and alcohol consumption, as well air pollution exposure in the same season. The impacts of macroeconomic and environmental factors on young men and women are manifested in greater rates of hospitalization for hypertension and IHD with a latent period of at least three months. The impacts of unemployment on young men and women are manifested in greater rates of hospitalization for IHD with a latent period of half a year. Finally, after controlling for unemployment and other economic factors, this study found that GDP exhibits no relevance to hospitalization rates for cardiovascular diseases.

## Supporting information

S1 File Input variable data. (XLSX)
